# Using Shockwave Lithotripsy to Treat Severe Calcified Mitral Stenosis in a Pregnant Woman

**DOI:** 10.1002/ccd.31641

**Published:** 2025-06-02

**Authors:** Mhd Baraa Habib, Cheikh Ahmed Aboulmaaly, Nazar Mohammed, Mohammed Al‐Hijji

**Affiliations:** ^1^ Department of Cardiology Heart Hospital, Hamad Medical Corporation Doha Qatar

**Keywords:** angiographic/fluoroscopic, IAF ‐ imaging, MVD ‐ mitral valve disease, MVPI ‐ mitral valve disease, percutaneous intervention, PREG ‐pregnancy

## Abstract

Rheumatic mitral stenosis (MS) with significant calcification presents challenges for both surgical and transcatheter interventions. Percutaneous balloon mitral valvuloplasty (PBMV) is often limited in these cases due to valve rigidity, increasing the risk of mitral regurgitation (MR). Transcatheter mitral valve lithotripsy (TMVL) is an emerging technique that uses sonic waves to disrupt calcification, enhancing valve pliability and PBMV outcomes. A 39‐year‐old woman, 18 weeks pregnant, with a history of rheumatic heart disease and prior surgical mitral commissurotomy, presented with palpitations, fatigue, and exertional breathlessness. She had severe MS (mitral valve area 0.8 cm²) and severe tricuspid regurgitation, with atrial fibrillation and rapid ventricular response. Due to a high Wilkins score, PBMV was attempted with a 28 mm Inoue balloon inflated to 28 mm, but was suboptimal due to significant valve rigidity. Adjunctive TMVL improved valve pliability, successfully reducing the mitral gradient and increasing valve area without worsening MR. The patient continued her pregnancy without complications. This case highlights TMVL as a promising adjunct to PBMV in severe calcified MS, particularly in high‐risk patients such as pregnant women. Further studies are needed to validate its efficacy and long‐term outcomes.

## 1 Introduction

Percutaneous balloon mitral valvuloplasty (PBMV) is the preferred treatment for rheumatic mitral stenosis (MS) when there is minimal calcification and a low Wilkins score. However, heavily calcified valves present significant risks, particularly for patients with high surgical risk [1].

Despite some reports indicating no harm when shockwave lithotripsy (SWL) was accidentally used during pregnancy, it is not part of current clinical practice due to potential risks to the fetus [2].

We successfully employed transcatheter mitral valve lithotripsy (TMVL) to enhance PBMV outcomes in a pregnant patient with severe calcified rheumatic MS, effectively addressing this complex issue.

## 2 Case Report

**Figure 1:**
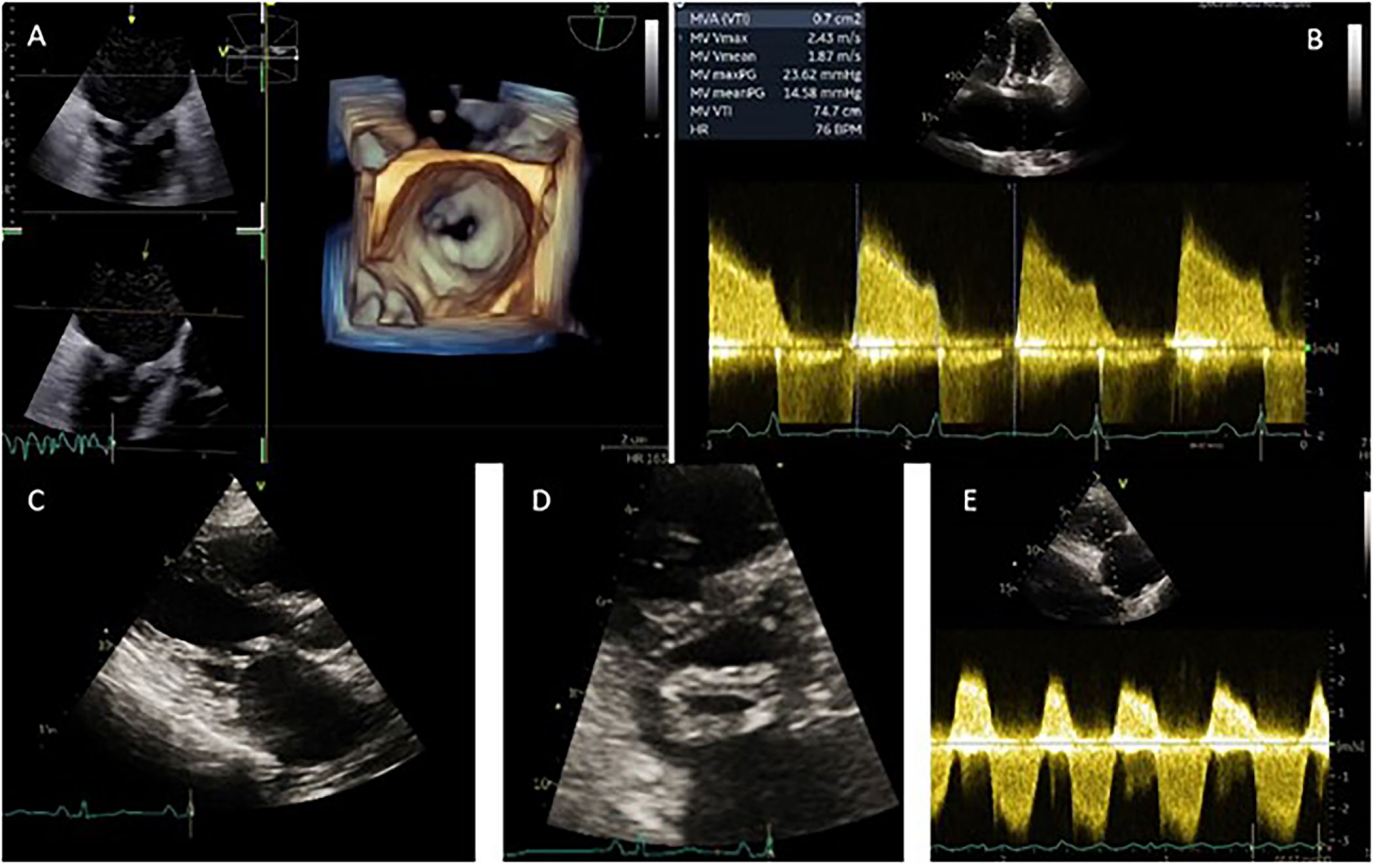
A pre‐procedure echocardiogram shows severe mitral valve stenosis with a mitral valve area of 0.8 cm² (3D‐image [A], 2D‐image [C, D]). Mitral valve mean gradient of 15 mmHg (B). Severe tricuspid regurgitation with right ventricular systolic pressure of 56 mmHg (E). [Color figure can be viewed at wileyonlinelibrary.com]

**Figure 2:**
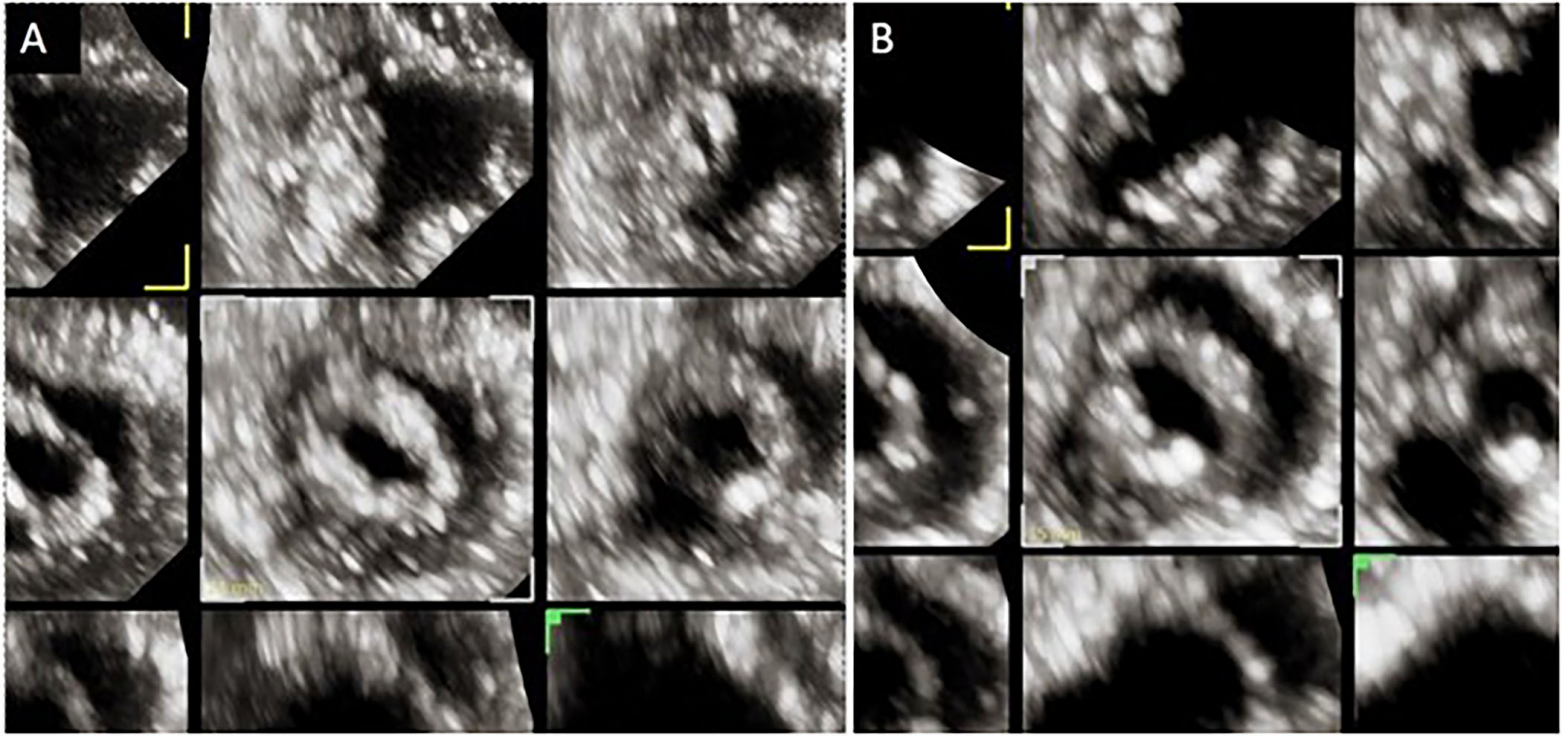
Transthoracic echocardiography—short‐axis view of the mitral valve after the initial Inque balloon inflation (A) and lithotripsy‐assisted balloon inflation (B). [Color figure can be viewed at wileyonlinelibrary.com]

A 39‐year‐old woman, 18 weeks pregnant, with a history of two recent abortions, rheumatic heart disease diagnosed over 20 years ago and previously treated with surgical mitral valve commissurotomy, presented with palpitations, fatigue, and exertional breathlessness for a few days. Her ECG indicated atrial fibrillation with rapid ventricular conduction. A transesophageal echocardiogram revealed severe mitral valve stenosis (MS) with a mitral valve area of 0.8 m², and MV mean gradient of 15 mmHg, mild aortic valve stenosis and regurgitation, and severe tricuspid valve regurgitation with right ventricular systolic pressure of 56 mmHg (Figures 1 and 2). Despite electrical cardioversion, restoring the sinus rhythm, controlling her heart rate, and diuresis, the patient remained breathless.

Considering the patient's symptoms, severe MS, pulmonary hypertension, and pregnancy, the case was discussed in a multidisciplinary meeting involving an obstetrician, cardiologist, interventional cardiologist, an anesthesiologist, and a cardiac surgeon. After the Heart Team's review and consultation with the patient, it was decided to proceed with a high‐risk PBMV as she refused elective abortion. However, given the patient's high Wilkins score of 10, indicating significant calcium buildup on the mitral valve and unfavorable anatomy, there were concerns about the effectiveness of traditional balloon valvuloplasty. However, due to the success reported in previous cases, the team opted for PBMV with back‐up of mitral valve SWL balloon valvuloplasty to address the calcium burden.

**Figure 3:**
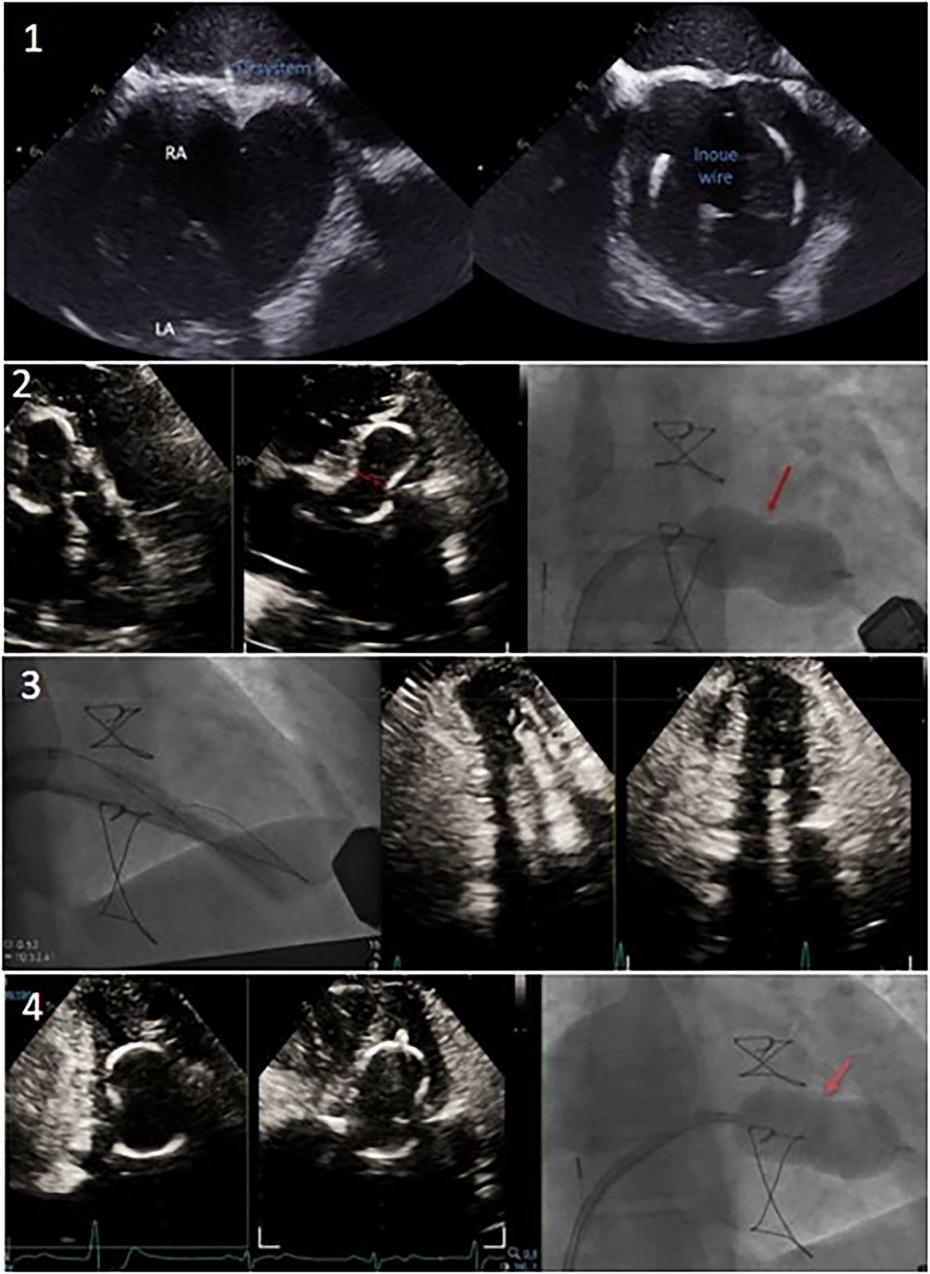
Step‐by‐step procedure. Step 1: Intracardiac echocardiogram ICE‐guided transseptal puncture, septal dilation, and delivery of a 28 mm Inoue balloon. Step 2: Multiple inflations with the 28 mm Inoue balloon, with failure to split the commissures. Step 3: Upsizing access to 22 Fr and delivering two 8.5 Fr steerable sheaths into the left atrium (LA) for simultaneous inflation of two 8.5 × 60 mm TMVL shockwave balloons. Step 4: Reinflation with the 28 mm Inoue balloon following TMVL treatment, achieving good balloon expansion. [Color figure can be viewed at wileyonlinelibrary.com]

**Figure 4:**
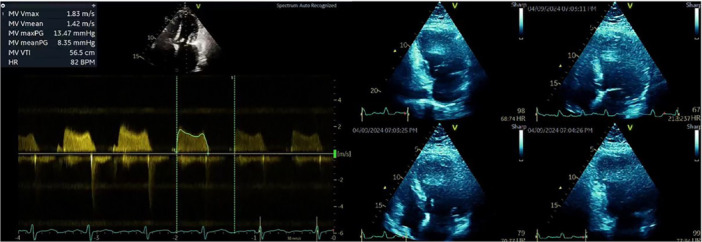
A postprocedure transthoracic echocardiogram. The MV mean gradient of 8 mmHg and improving the valve area to 1.5 cm^2^. [Color figure can be viewed at wileyonlinelibrary.com]

Local anesthesia was used to minimize the risk of general anesthesia during pregnancy. The catheterization table was covered with a led apron at the abdominal area of the patient and patient's abdomen was further covered with RadPad to maximally minimize radiation dose. The fluoroscopy frame rate was reduced to 4 frames per second. The interatrial septal puncture was established using a 21 G Brockenbrough needle and an 8 F Mullens sheath through the right femoral vein, with limited fluoroscopy time and with intracardiac echocardiography guidance (ICE) (Figure 3). The septum was dilated using a 14 F dilator over an LA wire, and a 28 mm Inoue balloon was passed across the mitral valve ring. Despite two inflations under ICE catheter guidance, commissural splitting was not achieved, and the gradient remained high. The access site was upsized to 22 Fr × 33 cm dryseal. Through the dryseal, two Agilis steerable sheaths were delivered to the left atrium over two safari wires. The valve was crossed with two multipurpose (MP) catheters, and 300 cm BMW 0.014 wires were placed. MP catheters were exchanged with two 8 mm TMVL balloons which were simultaneously inflated at 6 atm and sonic waves were delivered for three cycles (Figure 3). A broken BMW wire shaft was then noticed in the left ventricle, with its tip in the carotid artery. Access was obtained through the left femoral artery, and the wire was successfully snared from the ascending aorta with an AL‐1 6 Fr and en‐snare system. The BMW wires were exchanged with two ASAHI Astato XS 20 wires, sonic waves were delivered using two Shockwave 8.0 mm × 60 mm balloons for an additional eight cycles (Figure 3). Final ballooning with a 28 mm Inoue balloon to 28 mm resulted in excellent outcomes, reducing the gradient from 15 mmHg (by TEE) to 8 mmHg and improving the valve area from 0.6 to 1.5 cm² (Figures 2 and 4). Hemostasis in right femoral vein access was achieved with a figure‐of‐eight stitch, and the left femoral artery access with 8 Fr AngioSeal. The final radiation dose 0.18 Gycm² and fluoroscopy time was 19.3 min.

The patient was observed for 3 days after the procedure. Immediate fetal ultrasound showed normal fetal signs. She was clinically fine with no remarkable symptoms. She was discharged on oral Metoprolol 25 mg BID and Enoxaparin SC 70 mg BID. Over the next few weeks, clinical follow‐up showed a stable patient and fetus without cardiovascular symptoms. At 37 weeks, she had a vaginal delivery of a healthy baby.

## 3 Discussion

The 2021 ESC Guidelines recommend PBMV for patients with symptomatic severe MS or with systolic pulmonary artery pressure (SPAP) over 50 mmHg [3]. Calcific MS is particularly challenging for surgical treatment and is often not suitable for most modern transcatheter mitral valve replacement devices, leaving patients with few treatment options [1].

Mitral Valve SWL is a new technique used in a limited number of cases to improve pliability of calcification in the mitral valve, especially in patients with high Wilkins scores where traditional balloon valvuloplasty may be less effective [4, 5, 6, 7, 8, 9, 10]. It is hypothesized that SWL can fracture thick valvular calcium, allowing more expansion of the valvuloplasty balloon and making the calcified mitral leaflet tips more pliable to prevent significant mitral regurgitation after the procedure [4].

A review of 15 patients treated with TMVL for symptomatic calcific MS at St. Michael's Hospital, Canada, between 2021 and 2023, showed a significant reduction in mean trans‐mitral gradient and improved symptoms for 93% of patients, with no major complications at 90‐day follow‐up [6].

We report the first case of using SWL to facilitate PBMV in a pregnant patient. This case demonstrates the potential utility of using this technology to manage calcific MS during pregnancy. However, more data on a larger population is needed to evaluate its efficacy and safety further.

## 4 Conclusion

TMVL can enhance the effectiveness of PBMV in patients with severe calcified MS. This case has shown that TMVL might be safe and effective in a pregnant patient.

## Disclosure

The authors have nothing to report.

## Consent

The authors confirm that written consent for submission and publication of this case report has been obtained from the patient in line with COPE guidelines.

## Conflicts of Interest

The authors declare no conflicts of interest.

## Data Availability

The data that supports the findings of this study are available in the supplementary material of this article.
